# Faster steroid-free remission with tocilizumab compared to methotrexate in giant cell arteritis: a real-life experience in two reference centres

**DOI:** 10.1007/s11739-024-03722-4

**Published:** 2024-08-02

**Authors:** Luca Quartuccio, Elena Treppo, Maria De Martino, Maria Pillon, Simone Perniola, Dario Bruno, Miriam Isola, Elisa Gremese

**Affiliations:** 1 Rheumatology Division, Department of Medicine (DMED), University of Udine, Udine, Italy; 2 Institute of Statistics, Department of Medicine (DMED), University of Udine, Udine, Italy; 3 Division of Rheumatology, Institute of Rheumatology and Affine Sciences, School of Medicine, Catholic University of the Sacred Heart, Rome, Italy

**Keywords:** Giant cell arteritis, Tocilizumab, Methotrexate, Glucocorticoids, DMARDs, Steroid sparing

## Abstract

Glucocorticoids (GCs) are still the mainstay of treatment of giant cell arteritis (GCA). Although GCs are highly effective in GCA, the high burden of toxicity of GCs as well as the disease relapse during GC tapering is well documented. To compare the efficacy and rapidity of TCZ and MTX as steroid-sparing agents in a real-life cohort of GCA patients. A retrospective analysis was conducted including patients with newly diagnosed GCA from the Rheumatology Units of Udine and Rome. The inclusion criterion was the treatment with TCZ or MTX as first steroid-sparing drug. 112 GCA patients (81 females) with a median age of 70 (IQ 65–75) years were collected. Thirty-one out of 112 (27.7%) patients were treated with TCZ (162 mg/week), while 81/112 (72.3%) patients received MTX (up to 20 mg/week) as a GC-sparing agent. At month 6 after GCA onset, 5/31 (16.1%) patients in TCZ group and none in MTX group were in GC-free sustained remission (*p* value = 0.001). Similarly, at month 12, 64.5% (20/31) and 11.1% (9/81) of patients were in sustained GC-free remission in TCZ and MTX group, respectively (*p* value <0.001). At month 24 of follow-up, at least one relapse of the disease occurred in 7/31 (22.6%) in TCZ-treated and 28/81 (34.6%) in MTX-treated patients, respectively (*p* value = 0.22). TCZ allowed a faster discontinuation of steroid therapy than MTX in GCA patients, without increasing the risk of relapse.

## Introduction

Giant cell arteritis (GCA), also known as temporal arteritis or Horton disease, is an inflammatory disease of medium to large-sized vessels that can commonly involve cranial (C-), i.e. the classical GCA, and extra-cranial vessels, i.e. the large-vessel GCA (LV-GCA) [1]. GCA is the most common primary systemic vasculitis of the Western world in people older than 50 years of age, with a higher incidence in Northern Europe than in Southern Europe [2]. In approximately half of the cases, GCA is associated with polymyalgia rheumatica (PMR) [3] and the lifetime risk of developing GCA is higher in females than in males. The most feared and severe complications are blindness, in the short term, and aortic aneurism, in the long term.

Glucocorticoids (GCs) are still the mainstay of GCA treatment and guidelines recommend an initial dosage of 40–60 mg/day prednisone equivalent (PN-eq), followed by a gradual reduction over 1–2 years [4]. Although GCs are highly effective in controlling the disease, the high burden of toxicity of GCs as well as the disease relapse during GC tapering is well documented [5]. Therefore, patients with GCA may benefit from GC-sparing treatments [6]. Tocilizumab (TCZ) has been licensed as the first biologic treatment for GCA [7], and many other therapies have been used [e.g., methotrexate (MTX), cyclophosphamide [8, 9]] or are currently under investigation [10].

The aim of this retrospective study was to compare the efficacy and rapidity of TCZ and MTX as steroid-sparing agents in a real-life cohort of GCA patients.

## Methods

### Patient selection

We retrospectively enrolled patients affected by newly diagnosed GCA between 2003 and 2022, belonging to Rheumatology Units located in Udine and Rome. The diagnosis of GCA was based on the presence of symptoms and signs of GCA confirmed by positivity of at least one of the following exams: temporal artery biopsy (TAB), temporal artery ultrasound (US), or positron emission tomography and computed tomographic (PET/CT) imaging.

Inclusion criteria were: (1) clinical diagnosis of cranial (C-) or large-vessel (LV-) GCA confirmed by imaging or TAB and (2) treatment with TCZ or MTX as first steroid-sparing drug. Patients who were treated, as first Disease-Modifying Anti-Rheumatic Drug (DMARD), with TCZ, were included in TCZ-group; conversely, patients who were treated as first DMARD with MTX, were included in MTX-group.

Exclusion criteria were: (1) use of any other conventional synthetic (cs-) or biological (b-) DMARDs and (2) the presence of any other disease that could influence the request of GCs therapy.

Remission was defined as the absence of symptoms and signs of GCA documented by expert physician, along with the normalization of C-reactive protein (CRP) and erythrocyte sedimentation rate (ESR). Relapse of disease was defined as the recurrence of at least one symptom and/or sign (with or without an increase in the laboratory parameters of systemic inflammation) of GCA after achieving remission status. The steroid treatment begins with an initial high-dose phase of GC at 40–60 mg/day of PN-eq, except for patients with visual impairment who have received pulse GC therapy. Following this, a gradual tapering phase is implemented based on the physician’s evaluation.

Patients’ data were collected in a retrospective manner from our institution’s electronic medical records, including demographic characteristics, clinical manifestations, laboratory, and imaging results. We also collected any reported adverse event related to GC-therapy and steroid-sparing drugs, as well as tapering and discontinuation of GCs.

### Endpoints

The primary endpoint was to compare the percentage of patients with sustained GC-free remission at month 6 and month 12 in the TCZ group versus MTX group. The secondary endpoints were the assessment of the median time to achieve 7.5 mg and 5 mg PN-eq, and the median time to discontinuation of the steroid.

### Statistical analysis

Descriptive statistics are used to summarize the baseline characteristics of the study. Continuous data are reported as medians with interquartile range (IQR) or mean with standard deviation (SD). Categorical data are reported as counts with percentages. Comparisons between MTX- and TCZ-group were made by parametric (t-test for two independent samples) or no parametric (Mann–Whitney test for continuous variables; chi square tests for dichotomic variables). The level of significance used was *p* < 0.05.

### Compliance with ethical standards

All the procedures contributing to this work comply with the ethical standards of the relevant national and institutional committees on human experimentation and the Helsinki Declaration of 1975 as revised in 2008. This article does not contain any studies of human or animal subjects performed by any of the authors. Since this analysis was based on electronic clinical chart records, patients admitted to our hospital were asked to sign an informed consent for using their data for research purposes. The study was approved by the Department of Medicine Institutional Review Board (IRB) (Prot. 169/2022).

## Results

### Patients at baseline

From April 2003 to March 2022, we collected data on 112 consecutive GCA patients (81 females) who fulfilled the inclusion criteria. The median age at onset of disease was 70 (IQR 65–75) years; 31/112 (27.7%) patients and 81/112 (72.3%) patients were treated, as first DMARD, with TCZ and MTX, respectively. The baseline clinical characteristics of the two groups are reported in Table 1. MTX up to 20 mg/week was employed in 46/81 (56.8%) patients within 3 months of diagnosis, similarly, 19/31 (61.3%) patients of TCZ group received TCZ at the dose of 162 mg/week within 3 months of diagnosis. All patients received induction therapy with high-dose GC except one who, due to multiple concomitant comorbidities, received only TCZ immediately. The median time of follow up in the TCZ and MTX group was 41 (IQR 27–49) and 67 (IQR 42–99) months (*p* < 0.001), respectively. The age and gender distribution, as well as pre-existing comorbidities, were comparable between the groups (Table 1). Clinical symptoms at baseline such as visual impairment, jaw claudication, fever and PMR did not appear different (Table 2). Conversely, higher value of CRP at onset of disease were noticed in the TCZ group (*p* value = 0.037), whereas patients complaining of headache were statistically more represented in the MTX group (*p* value = 0.038).

**Table 1:** Baseline characteristics of 112 newly diagnosed GCA patients

	MTX (*n* = 81)	TCZ (*n* = 31)	*p* value
Age, median (IQR) (years)	71 (66–75)	68 (59–75)	0.367
Female, *n* (%)	61 (75.3)	20 (64.5)	0.253
ESR, median (IQR) (mm/h)	74 (57–92)	87 (56–105)	0.053
CRP, median (IQR) (mg/L)	49.9 (35–102)	79 (43.8–152)	**0.037**
Fibrinogen, median (IQR) (mg/dL)	763.5 (680–840)	723 (433–855)	0.455
TAB positive, *n*/*N* (%)	43/49 (87.8)	12/14 (85.7)	0.840
Halo sign positive, *n*/*N* (%)	9/13 (69.2)	8/13 (61.5)	1.000
PET/TC positive, *n*/*N* (%)	38/51 (74.5)	20/26 (76.9)	0.816
Comorbidities, *n* (%)			
Diabetes mellitus	7 (8.6)	4 (12.9)	0.493
Ischaemic heart disease	2 (2.5)	2 (6.5)	0.306
Arterial hypertension	38 (46.9)	15 (48.4)	0.889
Obesity	9 (11.1)	4 (12.9)	0.791
Osteoporosis	13 (16.1)	9 (30)	0.102
Previous malignancies	10 (12.4)	6 (19.4)	0.343
Presence of at least one risk factor^a^	52 (64.2)	21 (67.7)	0.725

*TCZ* tocilizumab, *MTX* methotrexate, *ESR* erythrocyte sedimentation rate, *CRP* C-reactive protein, *ACR* American College Rheumatology, *PMR* polymyalgia rheumatica, *TAB* temporal arterial biopsy, *PET/TC* positron emission tomography and computed tomographic

^a^Presence of at least one among diabetes mellitus, hypertension, ischaemic heart disease, obesity, or osteoporosis

Bold value indicates statistically significant comparisons

**Table 2:** Clinical presentation at onset of disease in the 112 newly diagnosed GCA patients

	MTX (*n* = 81)	TCZ (*n* = 31)	*p* value
Headache, *n* (%)	67 (87.7)	20 (64.5)	**0.038**
Temporal artery abnormalities, *n* (%)	44 (54.3)	14 (45.2)	0.385
Transient ocular symptoms, *n* (%)	25 (30.9)	9 (29)	0.850
Ocular complication, *n* (%)	8 (9.9)	6 (19.4)	0.206
Jaw claudication, *n* (%)	37 (45.7)	9 (29)	0.109
Extra-cranial symptoms, *n* (%)	44 (54.3)	18 (58.1)	0.721
Fever, *n* (%)	42 (51.9)	20 (64.5)	0.228
PMR, *n* (%)	41 (50.6)	11 (35.5)	0.151

*AION* anterior ischemic optic neuropathy, *PMR* polymyalgia rheumatica

Bold value indicates statistically significant comparisons

### Primary and secondary outcomes

**Fig. 1:**
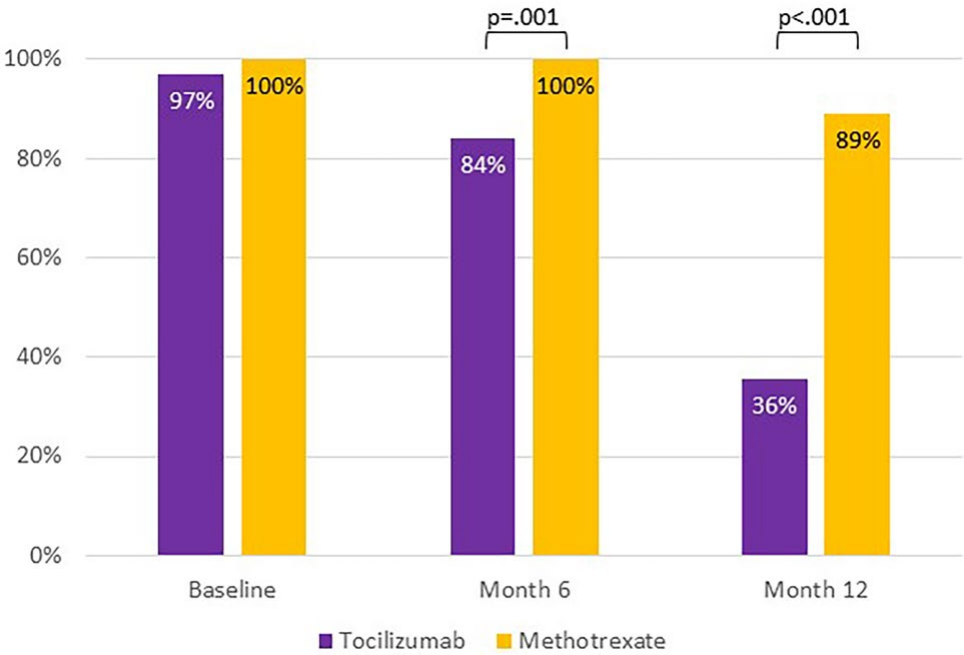
Percentage of patients on steroid therapy in the two groups at month 6 and month 12

One-hundred-and-ten out of 112 (98.2%) patients were in clinical remission at the last follow up. Overall, 25 out 31 (80.6%) patients in TCZ group and 60/81 (74.1%) patients in MTX group were on steroid-free therapy. The percentage of patients with sustained GC-free remission at month 6 was 16.1% (5/31) and 0% (0/81) in the TCZ group and the MTX group, respectively (*p* value = 0.001). Similarly, the percentage of patients with sustained GC-free remission at month 12 was 64.5% (20/31) and 11.1% (9/81) in the TCZ group and the MTX group, respectively (*p* value <0.001) (Fig. 1). The median time to achieve 7.5 mg and 5 mg PN-eq per day also differed in the two groups: the TCZ group achieved 7.5 mg PN-eq and 5 mg PN-eq at 4 (IQR 3–5) and 6 (IQR 5–7) months, respectively; conversely, the MTX group achieved the same dosage of GCs at 6 (IQR 4–9) and 9 (IQR 6–12) months, respectively. The median time to discontinued GCs was 10 (IQR 7–12) months (TCZ group) and 24 (IQR 18–45) months (MTX group) (*p* value <0.001). These results are shown in Table 3.

**Table 3:** Reduction and suspension of glucocorticoid therapy

	MTX (*n* = 81)	TCZ (*n* = 31)	*p* value
GC-free remission within 6 months, *n* (%)	0	5 (16.1)	**0.001**
GC-free remission within 12 months, *n* (%)	9 (11.1)	20 (64.5)	**<0.001**
Months to achieve 7.5 mg PN-eq (IQR)	6 (4–9)	4 (3–5)	**0.002**
Months to achieve 5 mg PN-eq (IQR)	9 (6–12)	6 (5–7)	**<0.001**
Months to achieve 0 mg PN-eq (IQR)	24 (18–45)	10 (7–12)	**<0.001**

*TCZ* tocilizumab, *MTX* methotrexate, *GC* glucocorticoid, *PN-eq* prednisone-equivalent

Bold values indicate statistically significant comparisons

To mitigate potential confounding factors associated with the study’s timeframe, a sub-analysis was conducted focusing on patients treated subsequent to the publication of the GiACTA study (i.e., during the period 2018–2022). Within this sub-analysis, a comparison was drawn between 27 patients receiving TCZ treatment and 22 patients receiving MTX treatment. The percentage of patients with sustained GC-free remission at month 6 was 18.5% (5/27) and 0% (0/22) in the TCZ group and the MTX group, respectively (*p* value = 0.056). Similarly, the percentage of patients with sustained GC-free remission at month 12 was 74.1% (20/27) and 22.7% (5/22) in the TCZ group and the MTX group, respectively (*p* value <0.001). The median time to achieve 5 mg PN-eq per day was 6 (IQR 5–6) months in TCZ group and 8 (IQR 6–12) months in MTX group (*p* value = 0.005). The median time to discontinued GCs was 9 (IQR 7–12) months (TCZ group) and 18 (IQR 11–20) months (MTX group) (*p* value <0.001).

### Relapses and safety of treatments

**Fig. 2:**
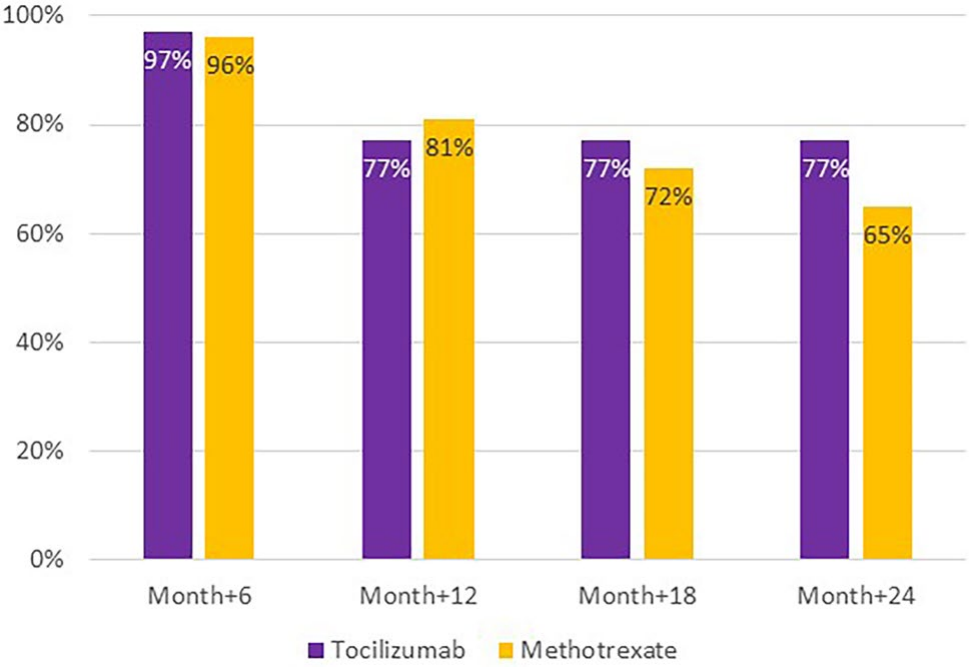
Percentage of relapse-free patients in the two groups from disease onset to month 24

At 24 months from onset of disease, at least one relapse of the disease occurred in 7/31 (22.6%) in TCZ-treated and 28/81 (34.6%) in MTX-treated patients, respectively (*p* value = 0.22) (Fig. 2). Overall, during the entire observation period considered, 62 relapses in 50 patients were registered: in about one-third of the cases (19/62) a transient increase of the steroid dose was sufficient, while 43/62 (69.4%) relapses required the initiation, reintroduction or combination of DMARDs.

Over the period of time considered, the retention rate was 77.4% (24/31) in TCZ group and 46.9% (38/81) in MTX group. Two patients of TCZ group switched to MTX due to adverse events (abdominal pain and difficulty swallowing), one patient required MTX to be added to TCZ, while 4 out of 31 were in remission without any DMARD (discontinuing TCZ after a median of 26.5 [IQR 23–29.75] months). On the other side, 15/81 (18.5%) patients of MTX group switched to TCZ due to adverse events (mainly nausea and general malaise) or relapses (8/15), and 28 out 81 had discontinued MTX after a median period of 40.5 (IQR 21–56.75) months. A total of 26/112 (23.2%) patients (13 in each group) repeated PET/CT scan with a median time of 18 months (IQR 12–24 months) showing improvement or absence of the disease.

At both the 12-month assessment and throughout the follow-up period, there was no statistically significant difference in the overall complication rate between the two groups (Table 4).

**Table 4:** Adverse events and outcomes in the two groups at 12 months and at last follow up

Adverse events	MTX group (*n* = 81)	TCZ group (*n* = 31)	*p* value
*At 12 months*			
Infection events requiring hospitalization	2 (2.5)	3 (9.7)	0.129
New-onset diabetes mellitus, *n* (%)	13 (16.1)	3 (9.7)	0.550
New-onset hypertension, *n* (%)	8 (9.9)	1 (3.2)	0.440
Secondary osteoporosis, *n* (%)	20 (24.7)	4 (12.9)	0.174
Fragility fractures, *n* (%)	8 (9.9)	1 (3.2)	0.440
Ischemic events, *n* (%)	3 (3.7)	1 (3.2)	1.000
Malignancies, *n* (%)	3 (3.7)	0	0.559
*At last follow-up*			
Infection events requiring hospitalization	2 (2.5)	3 (9.7)	0.129
New-onset diabetes mellitus, *n* (%)	13 (16.1)	3 (9.7)	0.550
New-onset hypertension, *n* (%)	9 (11.1)	1 (3.2)	0.279
Secondary osteoporosis, *n* (%)	23 (28.4)	4 (12.9)	0.086
Fragility fractures, *n* (%)	11 (13.6)	2 (6.5)	0.510
Ischemic events, *n* (%)	5 (6.2)	1 (3.2)	1.000
Malignancies, *n* (%)	6 (7.4)	0	0.184

*TCZ* tocilizumab, *MTX* methotrexate

## Discussion

European League Against Rheumatism (EULAR) recommendations [4] assert to add GC-sparing therapies for relapsing GCA patients or patients who have an increased risk of developing GC-related comorbidities. However, there is a trade-off to consider: a rapid reduction in GC dosage is linked to higher risk of relapses, while a delayed reduction in GC dose can result in a greater burden of side effects [11]. MTX and TCZ are the most commonly prescribed drugs for steroid-sparing treatment in practice [12] and there have been still no direct studies comparing treatment with MTX and TCZ in GCA.

This is the largest study that compared TCZ and MTX in a routine management of patients with GCA. In our study, 85 out of 112 consecutive patients (75.9%) discontinued GCs, and treatment with TCZ allowed a faster discontinuation of GCs than MTX (10 months versus 24 months, *p* value <0.001). Two thirds of the patients in TCZ group were in steroid-free remission within 12 months from disease onset (*p* value <0.001) (Fig. 1). Similarly, a higher steroid discontinuation rate has recently been demonstrated in PMR and in LV-GCA with TCZ, compared to MTX and GC alone [13, 14]. The RIGA study included 88 LV-GCA patients treated with either GC monotherapy, MTX combination, or TCZ combination. The results showed a decrease in PET vascular activity score (PETVAS) in the overall population, with TCZ treatment exhibiting stronger GC sparing effects compared to MTX [14]. In a previous meta-analysis, Mahr et al. [15] demonstrated a modest role of MTX (10–15 mg/week) in reducing GCA relapse frequency and total GC dose. In particular, MTX resulted in a cumulative steroid dose saving of 842 mg at week 48. In GiACTA trial [7], comparing patients in the TCZ arm with patients in the placebo arm who received the same 26-week prednisone taper, the total cumulative prednisone dose over the 52 weeks was 1862 mg and 3296 mg, respectively. The appropriate duration of GC therapy in GCA varies among studies, and a significant proportion of patients with GCA requires long-term GC treatment, sometimes indefinitely [16]. Comorbidity in GCA has been clearly linked to GC [17], and the toxicity of GC is largely dependent on its cumulative dose [18, 19], therefore it is important to use GC at the lowest effective dosage and for the shortest period of time. A recent meta-analysis demonstrated an overall prevalence of relapses of around 47% in patients treated with GC monotherapy [20]. The relapse rate was associated with shorter GC regimens [20], and the recurrences usually occurred during the first 12–24 months after GC discontinuation [16]. In our study, physicians seemed more confident in discontinuing the steroid during TCZ therapy; moreover, our results also confirmed the data in the literature, where about one-third of the patients are steroid dependent and they cannot suspend GCs [21].

Our study also provides an overview of the evolution of the GCA treatment landscape, particularly before and after the publication of the GiACTA study, resulting in the increased use of TCZ in the recent years. Our finding remained consistent and robust also when considering the sub-group of patients treated after 2018, and the faster discontinuation of GCs in TCZ group was not associated with an increased relapse risk (*p* value 0.22). In GiACTA study [7], 23% of patients who received TCZ weekly had a flare of the disease compared with 68% of patients in the placebo group; whereas in an open-label cross-sectional study [22], MTX decreased the chance of getting a relapse from 65 to 34%. In another case–control study [23], adding MTX in patients with high frequency of relapse resulted in a threefold reduction in the frequency of relapses per 10 person-years. Therefore, both TCZ and MTX allowed lower relapse rates in GCA patients [21], anyway the estimation of the effect size of treatment and the computation of number of patients needed to treat to prevent one GCA relapse remain a challenge due to differences between clinical practice and trials as well as early and late initiation of DMARDs [6].

Long-term use of TCZ and MTX is generally safe and well tolerated [7, 15], and a retrospective real-life study supported the safety of a combined therapy (TCZ plus MTX) in refractory patients [24]. Furthermore, other studies confirmed their good safety profile even in older patient, which is a crucial feature in GCA [23, 25]. The main limitations to their use are related to renal function (for MTX) and a history of gastrointestinal perforations or diverticulitis (for TCZ) [21]. In our cohort, all treatment-related adverse events were mild and the discontinuation of the drug was always effective in resolving the event. The most common adverse effects were abdominal discomfort, nausea and general malaise. In addition, no differences were observed as concerns GC-related adverse events between the two groups. The relative risk of adverse events is estimated to increase by 3% for each exposure to 1000 mg prednisone [26]. In our study, we mainly recorded the new-onset of type 2 diabetes mellitus and secondary osteoporosis (±fragility fractures) as GC-related adverse events, with a trend towards a lower incidence of secondary osteoporosis under TCZ (*p* = 0.086).

The optimal duration of treatment with immunosuppressants (or the dose reduction in the follow up) remains an unmet need. A randomized controlled study [27] reported that the discontinuation of TCZ (after a 52-week treatment) led to a clinical relapse in about half of the patients. Moreover, the 3-year results from GiACTA study [28] showed that TCZ weekly was more effective than TCZ every-other-week in patients with relapsing disease. Undoubtedly, there is a need to better understand the risk factors for GCA relapse after treatment discontinuation.

### Study strengths and limitations

Our study has several strengths that enhance its relevance and applicability to real-world clinical practice. It provides valuable insights by allowing a comparison between the most commonly used therapies in GCA. The strengths of our study include the “real-life” nature, supporting existing literature data, and the enrolment of patients from two well-established reference centres for GCA, with experience in the diagnosis and treatment of systemic vasculitides, where a similar management of GCA patients has been already published [6].

The limitations of our study are related to the retrospective design and the small size of the cohort. Firstly, the considered timeframe includes the period before and after the publication of GiACTA study. The GiACTA study may have had an impact on GC tapering, potentially influencing a more confident reduction of GCs, nevertheless, our sub-analysis reinforces the robustness of our initial conclusions and underscores the strength of our results beyond the changing treatment paradigms brought about by the GiACTA study. Secondly, our study included both C-GCA and LV-GCA patients. This heterogeneity the population could cause differences in the disease features and severity, as well as in the response to the treatment, and to the overall outcomes. However, it is important to clarify that our study did not apply a standardized GC tapering schedule. Instead, the physicians tailored the treatment approach and management to each patient. The decision-making process regarding GC tapering was left to the discretion of the treating physician, who considered different factors, including disease severity, patient response, comorbidities, and safety concerns when adjusting the treatment regimen. This individualized approach really mirrors real-world clinical practice and captures the real-world complexities of GCA management.

## Conclusion

GCA is a chronic disease with high risk of recurrences and long-term complications related both to disease itself and to GC therapy. In real-world practice, physicians are used to add MTX or TCZ even at the beginning of the disease as steroid-sparing agents. In our study, TCZ allowed a faster discontinuation of steroid therapy without an increased relapse risk compared to MTX (Fig. 2). Long-term effects on GC-related comorbidity by these drugs deserve further studies, however, a better outcome with TCZ may be expected by our preliminary results. Although the optimal duration of treatment with immunosuppressants remains an unmet need, both drugs confirmed to be safe, well-tolerated even in elderly patients, when used in reference centres.

## Data Availability

Available.
